# Assessment of sepsis-induced immunosuppression at ICU discharge and 6 months after ICU discharge

**DOI:** 10.1186/s13613-017-0304-3

**Published:** 2017-08-02

**Authors:** Violette Zorio, Fabienne Venet, Benjamin Delwarde, Bernard Floccard, Guillaume Marcotte, Julien Textoris, Guillaume Monneret, Thomas Rimmelé

**Affiliations:** 1 Department of Anesthesiology and Critical Care Medicine, Edouard Herriot hospital, Hospices Civils de Lyon, Lyon, France; 2 EA7426 Hospices Civils de Lyon – bioMérieux, University Claude Bernard Lyon 1 “Pathophysiology of Injury Induced Immunosuppression”, Lyon, France; 3 Cellular Immunology Laboratory, Edouard Herriot hospital, Hospices Civils de Lyon, Lyon, France

**Keywords:** CD4+ T cells, HLA-DR, Immune system, Immunology, Immunosuppression, Regulatory T cells, Sepsis

## Abstract

**Background:**

Increase in mortality and in recurrent infections in the year following ICU discharge continues in survivors of septic shock, even after total clinical recovery from the initial septic event and its complications. This supports the hypothesis that sepsis could induce persistent long-term immune dysfunctions. To date, there is almost no data on ICU discharge and long-term evolution of sepsis-induced immunosuppression in septic shock survivors. The aim of this study was to assess the persistence of sepsis-induced immunosuppression by measuring expression of human leukocyte antigen DR on monocytes (mHLA-DR), CD4+ T cells, and regulatory T cells (Treg) at ICU discharge and 6 months after ICU discharge in patients admitted to the ICU for septic shock.

**Methods:**

In this prospective observational study, septic shock survivors with no preexisting immune suppression or treatment interfering with the immune system were included. mHLA-DR, CD4+ T cells, and Treg expression were assessed on day 1–2, 3–4, and 6–8 after ICU admission, at ICU discharge, and 6 months after ICU discharge.

**Results:**

A total of 40 patients were enrolled during their ICU stay: 21 males (52.5%) and 19 females, median age 68 years (IQR 58–77), median SOFA score on day 1–2 was 8 (IQR 7–9), and median ICU length of stay was 11 days (IQR 7–24). Among these 40 patients, 33 were studied at ICU discharge and 15 were disposed for blood sampling 6 months after ICU discharge. On day 1–2, mHLA-DR expression was abnormally low for all patients [median 4212 (IQR 2640–6047) AB/C] and remained abnormally low at ICU discharge for 75% of them [median 10,281 (IQR 7719–13,035) AB/C]. On day 3–4, 46% of patients presented CD4+ lymphopenia [median 515 (IQR 343–724) mm^−3^] versus 34% at ICU discharge [median 642 (IQR 459–846) mm^−3^]. Among patients with a 6-month blood sample, normal values of mHLA-DR were found for all patients [median 32,616 (IQR 24,918–38,738) AB/C] except for one and only another one presented CD4+ lymphopenia.

**Conclusions:**

While immune alterations persist at ICU discharge, there is, at cellular level, no persistent immune alterations among septic shock survivors analyzed 6 months after ICU discharge.

## Background

Despite progress in the therapeutic management of patients with sepsis and septic shock, hospital mortality of these patients has only slightly decreased in recent decades and remains around 20 and 40%, respectively [1]. While mortality in the first week has decreased, it has increased during subsequent weeks [2]. It is hypothesized that sepsis-induced immunosuppression occurring during this second phase of sepsis explains the “late” mortality, most likely due to increased risk of secondary infections [3].

Sepsis-induced immunosuppression is characterized by alterations affecting both innate and adaptive immunity. Among the biological parameters assessing the innate immune system, the level of the human leukocyte antigen DR expression on the surface of monocytes (mHLA-DR) is one of the best studied, and is recognized as an effective indicator of immunosuppression [4, 5]. Furthermore, a reduction in mHLA-DR is the only immune parameter shown to be an independent factor of nosocomial infection occurrence [6] and of mortality [7]. With regard to adaptive immunity, lymphopenia on the fourth day following the diagnosis of sepsis predicts mortality and may also serve as a biomarker for sepsis-induced immunosuppression [8]. Regulatory T cells (Treg) are less-well studied but present nevertheless well-defined and broad immunosuppressive properties [9–11]. Thus, in sepsis, the increase in the proportion of Treg cells among the total number of T CD4+ lymphocytes is correlated with poor prognosis [12].

Whereas the occurrence of immunosuppression during ICU stay is well documented, to date, there is almost no data on ICU discharge and on long-term evolution of sepsis-induced immunosuppression in survivors of septic shock. Some data support the hypothesis that sepsis could induce persistent long-term immune dysfunctions even long after the hospital discharge. Indeed, in the longer term, even after total clinical recovery in sepsis survivors, increase in mortality continues. It has been reported mortality of 43% after 1 year [13], 45% after 2 years [14], and 74% after 5 years [15] beyond hospital discharge. Furthermore, an increase of recurrent infections in the year following ICU discharge has also been reported in such patients [16, 17]. Taken together, persistent sepsis-induced immunosuppression at ICU discharge and in long term could be the underlying reason for this delayed mortality in sepsis survivors.

The aim of this study was to assess the persistence of sepsis-induced immunosuppression at the cellular level at ICU discharge and 6 months thereafter by measuring mHLA-DR, CD4+ T cells, and Treg expression in patients admitted for septic shock.

## Methods

### Study population

In this prospective cohort study, we included all patients who were admitted to the ICU for septic shock diagnosed for less than 48 h, and who were alive at the time of ICU discharge. The diagnosis of septic shock was retained in presence of sepsis associated with persistent hypotension despite adequate intravascular volume expansion and thus requiring initiation of vasopressors. We considered the diagnosis of septic shock when the required amount of vasopressors was greater than 0.25 μg/kg/min for at least 2 h. All patients under 18 years of age, pregnant women, as well as those with comorbidities related to the immune system or treatment interfering with the immune system were excluded (i.e., patients with hematological disease receiving or having received immunosuppressive drugs or immunomodulatory drugs within 5 years preceding inclusion, patients suffering from a solid tumor undergoing chemotherapy, from acquired aplasia, or from innate immune deficiency, patients who received extracorporeal life support (extracorporeal membrane oxygenation in context of acute respiratory distress syndrome or cardiogenic shock or extracorporeal circulation in relation to cardiac surgery) within 1 month prior to inclusion and patients treated with immunosuppressive drugs or corticosteroids at immunosuppressive doses.

### Ethical requirements

Each analyzable patient—if clinical conditions allowed it—or every corresponding reliable person was informed orally about objectives and conduct of the study and received a note summarizing this information. Patients were included only when the non-opposition had been expressed. For this non-interventional study assessing clinical data and biological samples, oral informed consent from the patient or a relative was considered sufficient. Written informed consent is not required by French law for studies carried out in current care. The biological data collection is reported to the ministry of research (Registered Under the Number DC-2008-509).

### Data collection

Patients were monitored clinically and biologically during their ICU stay. Blood samples were drawn: on day 1 or 2 (day 1–2), on day 3 or 4 (day 3–4), on day 6, 7, or 8 (day 6–8) of admission, and the day of ICU discharge (which could sometimes correspond to the day 6–8 sample, or more rarely to the day 3–4 sample for patients with rapid favorable outcome). Day 1 was the day of ICU admission. All survivors were asked to attend a consultation 6 months after ICU discharge, during which an additional blood sample was collected. Patients were contacted by telephone. Analyses included quantification of mHLA-DR at each time point and measurement of CD4+ T cells and Treg on day 3–4, at ICU discharge, and 6 months after ICU discharge. For each included patient, routine biological parameters and clinical data of ICU stay were collected. This 6-month post-ICU discharge consultation is usually offered to all patients admitted in our ICU irrespective of the reason for admission. During this medical consultation, we assess the psychological state of patients related to their ICU stay and we perform a medical examination.

### mHLA-DR, CD4+ T cells and Treg measurement

At each time point, samples were collected using EDTA tubes. All blood samples were stained within 1 h after collection or were immediately stored at 4 °C, in accordance with the standardization recommendations for mHLA-DR measurement [18, 19]. For mHLA-DR measurement, staining and cell acquisition by flow cytometry (FC500, Beckman Coulter, Hialeah, FL, USA) were performed as described in the European standardized protocol and according to the manufacturer’s recommendations (Quantibrite HLA-DR/Monocyte antibody cocktail, BD Bioscience, San Diego, CA, USA). Results were expressed as the number of anti-HLA-DR antibodies per cell (AB/C) (normal >15,000), which is correlated with the number of HLA-DR molecules expressed on each monocyte. CD4+ T cells were measured by flow cytometry based on expression of CD4; Treg cells were identified based on expression of CD4, CD25, and CD127, and results were expressed as the total number of Treg cells and as the proportion of the total number of CD4+ T cells.

### Statistical analysis

Data are expressed as median (IQR). Comparison of baseline characteristics between patients with a blood sample at 6 months and those without was done using the Fisher’s exact test or the Chi-square test, as appropriate. The Wilcoxon–Mann–Whitney’s test was used to determine differences between immunological variables at each time point. Pearson’s correlation coefficients were performed to study the correlation between immunological variables at ICU discharge and ICU length stay. Level of significance was set at 0.05. Calculations were performed using *R* statistical software [20].

## Results

### Study population

**Fig. 1:**
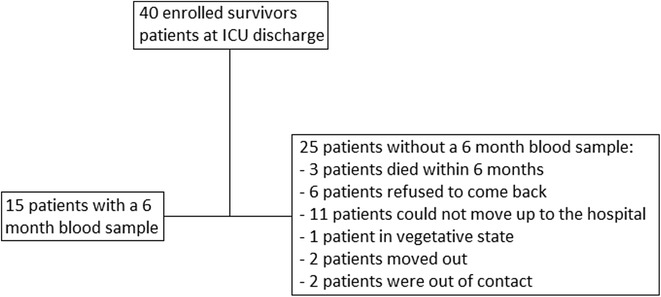
Study flowchart

From March 2015 to May 2016, 40 patients with septic shock were enrolled, and from October 2015 to November 2016, they were contacted by telephone. A total of 15 patients (37.5%) came back to hospital 6 months after their ICU discharge and consented to give blood (Fig. 1). Twenty-five patients did not come back to the hospital 6 months after their ICU discharge: three patients died within 6 months, one patient was in a vegetative state in another health institution, six patients refused to come back, 11 patients were willing to come but unable to travel to the hospital, two patients moved to cities too far for them to travel back, and two patients could not be contacted by telephone. Among the 11 patients unable to travel to the hospital, one patient did not come back to the hospital because he suffered from a lung infection.

There were 21 (52.5%) male patients, and the median (IQR) age was 68 (58–77) years (Table 1). Most patients (82.5% of the total study population) needed surgery in addition to antibiotics (Table 1). Six patients (15%) experienced a secondary nosocomial infection during their ICU stay: three lung infections, two surgical site infection, and one urinary tract infection. The characteristics of those with a blood sample at 6 months were similar to those without, with two exceptions: diabetes mellitus was not a comorbidity of those with a sample at 6 months but present in 8 (32%) of those without (*p* = 0.01), and stomatological focus was not a source of septic shock among those without but present in 3 (20.0%) of those with a sample (*p* = 0.04; Table 1).

**Table 1:** Patient characteristics

	Total study population *n* = 40	Patients with a blood sample at 6 months *n* = 15	Patients without a blood sample at 6 months *n* = 25	*p* value
Age (years), median (IQR)	68 (58–77)	62 (48–72)	71 (62–78)	0.08
Male gender, *n* (%)	21 (52.5)	8 (53.3)	13 (52.0)	1
SOFA score on day 1–2, median (IQR)	8 (7–9)	9 (7–10)	7 (6–9)	0.38
SAPS II, median (IQR)	53 (43–66)	52 (48–62)	53 (42–69)	0.71
Maximum lactate (mmol/L), median (IQR)	2.7 (1.8–3.7)	3.5 (2.5–5.9)	2.4 (1.7–2.9)	0.37
Missing data, *n*	3		3	
Comorbidities, *n* (%)				
Chronic arterial hypertension	21 (52.5)	6 (40.0)	15 (60.0)	0.32
Chronic heart failure	6 (15.0)	1 (6.7)	5 (20.0)	0.38
Myocardial infarction	3 (7.5)	1 (6.7)	2 (8.0)	1
Stroke	2 (5.0)	1 (6.7)	1 (4.0)	1
Chronic obstructive pulmonary disease	9 (22.5)	2 (13.3)	7 (28.0)	0.44
Dialysis-dependent chronic kidney disease	1 (2.5)	0	1 (4.0)	1
Liver cirrhosis	0	0	0	1
Diabetes mellitus	8 (20.0)	0	8 (32.0)	0.01
Source of infection, *n* (%)				
Abdominal	20 (50.0)	6 (40.0)	14 (56.0)	0.51
Skin and soft tissue	7 (17.5)	1 (6.7)	6 (24.0)	0.22
Respiratory	5 (12.5)	2 (13.3)	3 (12.0)	1
Stomatological	3 (7.5)	3 (20.0)	0	0.04
Urinary tract	3 (7.5)	2 (13.3)	1 (4.0)	0.54
Osteoarticular	2 (5.0)	1 (6.7)	1 (4.0)	1
General management, *n* (%)				
Surgery	33 (82.5)	13 (86.7)	20 (80.0)	0.69
Medical only	7 (17.5)	2 (13.3)	5 (20.0)	0.69
Support measures, *n* (%)				
Norepinephrine	40 (100.0)	15 (100.0)	25 (100.0)	1
Dobutamine	5 (12.5)	1 (6.7)	4 (16.0)	0.63
Mechanical ventilation	31 (77.5)	11 (73.3)	20 (80.0)	0.70
Renal replacement therapy	12 (30.0)	4 (26.7)	8 (32.0)	1
Outcomes (days), median (IQR)				
Days on vasopressors	4 (3–5)	4 (3–10)	3 (2–5)	0.30
Days on mechanical ventilation	6 (3–8)	7 (4–9)	5 (2–7)	0.43
Patients involved, *n*	31	11	20	
Days on renal replacement therapy	8 (6–8)	10 (8–12)	7 (4–8)	0.05
Patients involved, *n*	12	4	8	
ICU length of stay	11 (7–24)	13 (6–25)	10 (7–24)	0.86
Hospital length of stay	37 (16–48)	37 (19–46)	37 (19–49)	0.75

*IQR* interquartile range, *SOFA* Sepsis-related Organ Failure Assessment, *SAPS II* Simplified Acute Physiology Score II, *ICU* intensive care unit

There was no statistically significant difference for all routine biological and immunological parameters performed during the first week after admission between the population with a 6 months blood sample and those without (Table 2). Seventy-four per cent of the total population had lymphopenia (lymphocytes <1.5 mm^−3^ × 10^3^) on day 1–2 and this persisted in 56% of patients on day 6–8.

**Table 2:** Biological parameters

	Total study population *n* = 40	Patients with a blood sample at 6 months *n* = 15	Patients without a blood sample at 6 months *n* = 25	*p* value
Leukocytes (mm^−3^ × 10^3^), median (IQR)				
D1-2	16.6 (10.4–22.1)	15.3 (7.3–22.1)	16.6 (13.0–20.7)	0.41
Missing data, *n*	1	0	1	
D3-4	15.8 (10.1–21.3)	14.6 (10.6–21.5)	15.8 (9.7–19.7)	0.75
Missing data, *n*	9	1	8	
D6-8	14.2 (12.7–19.3)	13.8 (10.8–15.8)	15.4 (12.8–19.5)	0.88
Missing data, *n*	15	5	10	
Neutrophils (mm^−3^ × 10^3^), median (IQR)				
D1-2	13.7 (7.8–20.0)	13 (6.3–20.0)	13.9 (10.4–19.1)	0.47
Missing data, *n*	1	0	1	
D3-4	13 (8.8–18.5)	12.7 (9.5–18.9)	12.8 (8.4–16.3)	0.67
Missing data, *n*	9	1	8	
D6-8	11.8 (9.0–16.2)	11.6 (8.5–13.7)	12 (9.7–16.5)	0.76
Missing data, *n*	15	5	10	
Lymphocytes (mm^−3^ × 10^3^), median (IQR)				
D1-2	1 (0.5–1.5)	0.5 (0.3–0.9)	1.1 (0.7–1.8)	0.83
Missing data, *n*	1	0	1	
D3-4	1 (0.7–1.4)	1 (0.9–1.2)	1 (0.6–2.0)	0.99
Missing data, *n*	9	1	8	
D6-8	1.3 (1.0–2.2)	1.1 (1.0–1.3)	2 (1.0–2.2)	0.17
Missing data, *n*	15	5	10	
Monocytes (mm^−3^ × 10^3^), median (IQR)				
D1-2	0.5 (0.3–0.9)	0.4 (0.3–0.6)	0.6 (0.37–1.12)	0.78
Missing data, *n*	1	0	1	
D3-4	0.6 (0.4–0.9)	0.6 (0.4–0.7)	0.55 (0.42–1.0)	0.84
Missing data, *n*	9	1	8	
D6-8	0.7 (0.4–0.8)	0.4 (0.6–0.7)	0.7 (0.61–1.1)	0.35
Missing data, *n*	15	5	10	
mHLA-DR (AB/C), median (IQR)				
D1-2	4212 (2640–6047)	3321 (2246–6022)	4486 (2673–6047)	0.41
Missing data, *n*	4	3	1	
D3-4	4514 (3232–6497)	4989 (1484–7379)	4235 (3365–5555)	0.51
Missing data, *n*	1	1	0	
D6-8	7442 (4315–9724)	4817 (3059–9143)	7808 (5070–10,249)	0.13
Missing data, *n*	8	4	4	
ICU discharge	10,281 (7719–13,035)	10,228 (8719–11,826)	11,271 (7195–16,594)	0.54
Missing data, *n*	7	2	5	
6 months post-ICU discharge	32,616 (24,918–38,738)	32,616 (24,918–38,738)		
Missing data, *n*	25	0		
CD4+ T cells (mm^−3^), median (IQR)				
D3-4	515 (343–724)	477 (356–670)	537 (344–963)	0.63
Missing data, *n*	1	1	0	
ICU discharge	642 (459–846)	765 (459–796)	628 (463–979)	0.85
Missing data, *n*	11	6	5	
6 months post-ICU discharge	797 (647–1057)	797 (647–1057)		
Missing data, *n*	26	1		
Treg percentage, median (IQR)				
D3-4	6.6 (5.0–9.3)	7 (5.1–10.8)	6.4 (4.9–9.0)	0.54
Missing data, *n*	1	1	0	
ICU discharge	6.6 (5.2–7.9)	6.6 (5.0–8.3)	6.53 (5.5–7.6)	0.80
Missing data, *n*	11	6	5	
6 months post-ICU discharge	6.4 (4.7–8.0)	6.4 (4.7–8.0)		
Missing data, *n*	26	1		
Absolute Treg (mm^−3^), median (IQR)				
D3-4	35 (22–45)	34 (22–43)	35 (23–47)	0.92
Missing data, *n*	1	1	0	
ICU discharge	39 (28–64)	39 (28–64)	38 (28–77)	0.85
Missing data, *n*	11	6	5	
6 months post-ICU discharge	44 (34–67)	44 (34–67)		
Missing data, *n*	26	1		

*IQR* interquartile range, *AB/C* anti-HLA-DR antibodies per cell, *ICU* intensive care unit

### mHLA-DR expression

**Fig. 2:**
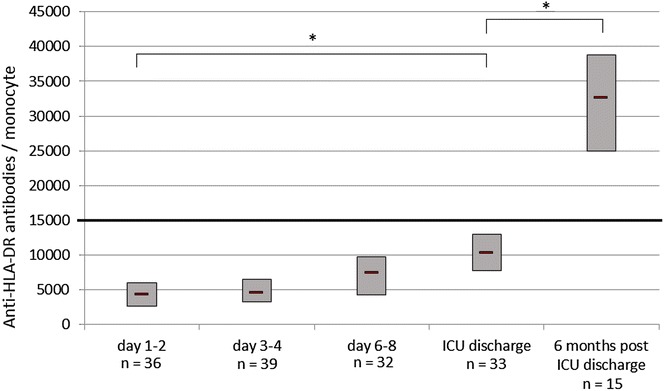
mHLA-DR expression from day 1 after admission to ICU discharge. Box plot interpretation: box borders: 1st and 3rd quartile, central band in box: median value, *significantly different

At admission, the median (IQR) mHLA-DR was 4212 (2640–6047) AB/C and remained <10,000 AB/C at day 6–8 (Table 2). All patients had abnormal mHLA-DR (<15,000 AB/C) either on day 1–2 or day 3–4. Two patients had normal mHLA-DR on day 6–8, whereas this was abnormal on day 3–4. At ICU discharge, eight patients (24%) had normal mHLA-DR. mHLA-DR was less than 5000 AB/C for 21 patients (58%) on day 1–2, for 22 patients (56%) on day 3–4, for 11 patients (34%) on day 6–8, and four patients (12%) at ICU discharge. For all patients, median mHLA-DR expression on day 1–2 was significantly lower to that at ICU discharge (*p* < 0.0001; Fig. 2). Three patients died within 6 months. One patient died from complications of incurable cytomegalovirus ileitis in a surgical unit 20 days after ICU discharge; for this patient mHLA-DR expression at ICU discharge was 9600 AB/C. Another patient died from an acute respiratory distress syndrome complicating lung infection in another critical care unit where he had been admitted 4 months after discharge from the study ICU; for this patient mHLA-DR expression at ICU discharge was 5555 AB/C. The last patient died at home 4 months after ICU discharge and the cause of death was cardiac arrest; for this patient mHLA-DR expression at ICU discharge was 28,447 AB/C. mHLA-DR expression on day 6–8 was not significantly different between those who had experienced a secondary nosocomial infection during ICU stay and those who had not done so (*p* = 0.19). mHLA-DR expression at ICU discharge was weakly correlated with the ICU length stay (*r* = 0.44, 95% CI 0.12–0.68, *p* = 0.01).

Among patients with a blood sample (*n* = 15), all but one patient had a normal level of mHLA-DR expression 6 months after ICU discharge (Fig. 2). The patient who had abnormal mHLA-DR expression suffered from recurrent abdominal sepsis requiring multiple surgical procedures since ICU discharge (29 days after his ICU admission) and was still hospitalized (in a surgical unit) 6 months after ICU discharge. For the 15 patients, median mHLA-DR expression on day 1–2 was significantly lower to that at ICU discharge (*p* = 0.005), and at 6 months after ICU discharge (*p* < 0.0001); median mHLA-DR expression 6 months after ICU discharge was significantly higher from median mHLA-DR at ICU discharge (*p* = 0.0002).

For the 11 patients who did not provide a blood sample at 6 months because they were unable to travel to the hospital, median mHLA-DR at ICU discharge was similar to those of the 15 with a blood sample at 6 months [median 11,497 (IQR 7195–21,032) AB/C vs. median 10,228 (IQR 8719–11,826) AB/C, respectively, *p* = 0.32].

### CD4+ T cells

**Fig. 3:**
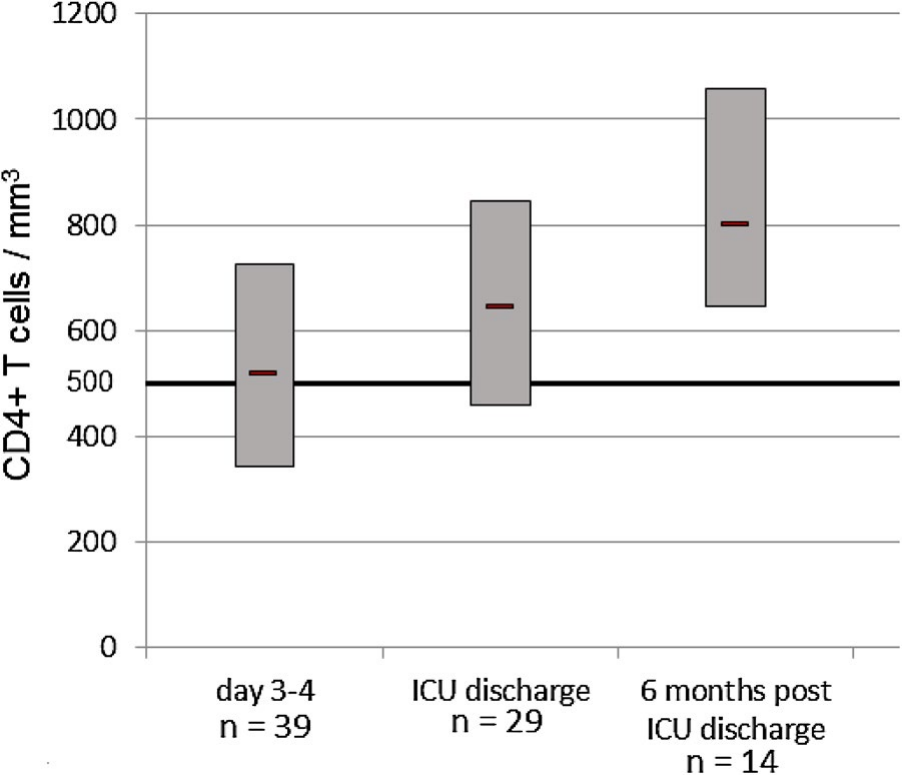
CD4+ T cells from day 3 after ICU admission to 6 months after ICU discharge. Box plot interpretation: box borders: 1st and 3rd quartile, central band in box: median value

Eighteen patients (46%) had CD4+ lymphopenia (<500 mm^−3^) on day 3–4 and 10 (34%) had CD4+ lymphopenia at discharge. The median (IQR) CD4+ T cells at ICU discharge was 642 (459–846) mm^−3^ (Table 2). Among the 15 patients with a blood sample at 6 months, only one patient presented CD4+ lymphopenia 6 months after ICU discharge (Fig. 3). This patient did not present a medical complication within this period. The number of CD4+ T cells at ICU discharge was not correlated with the ICU length of stay (*r* = 0.23, 95% CI −0.15 to 0.55, *p* = 0.22).

### Treg expression

The median (IQR) percentage of Treg relative to total CD4+ T cells at ICU discharge was 6.6 (5.2–7.9)% (Table 2). Seven patients out of the 39 (18%) with data available had increased proportion of Treg among CD4+ T cells (>10%) on day 3–4, whereas only 3 out of 29 (10%) did so at ICU discharge; this difference did not reach statistical significance (*p* = 0.49). All patients who had an elevated proportion of Treg also had mHLA-DR <5000 AB/C either on day 1–2 or on day 3–4. Two patients had a proportion of Treg greater than 15% on day 3–4. These two patients also had the highest Sepsis-related Organ Failure Assessment score (SOFA score) at admission. Among the 15 patients with a blood sample at 6 months, none had an abnormal proportion of Treg 6 months after ICU discharge.

## Discussion

This study found persistent cellular immune alterations in septic shock survivors at ICU discharge but no detectable long-term immune alterations 6 months after ICU discharge in some of them, as evidenced from mHLA-DR expression, CD4+ T cells, and relative proportion of Treg. Although unexpected, these data suggest that sepsis-induced immunosuppression at the cellular level based on these three markers is reversible after a period of time.

Among the biological parameters assessing the innate immune system, mHLA-DR seems to be an effective indicator of immune alterations [4, 5]. Its decreased expression represents one of the only immune marker shown to be an independent factor of occurrence of secondary nosocomial infections [6]. The interest of mHLA-DR expression assessment stands in the fact that this marker is a consequence of the effect of multiple mediators on the inflammatory response [21–23]. Its routine determination could be an important element of the immunological monitoring of septic patients [24, 25] or even of all patients undergoing an inflammatory injury and at risk of developing secondary infections. Usually, a patient is considered to be immunocompetent when mHLA-DR expression is above 15,000 AB/C. Values comprised between 10,000 and 15,000 AB/C may represent moderate immunosuppression, whereas values lower than 5000 AB/C may represent very deep immune alterations [18] which existed in 80% of patients in the present study, either on day 1–2 or on day 3–4. To our knowledge, there is no data in the medical literature assessing mHLA-DR expression at ICU discharge in patients admitted to the ICU for septic shock which would allow us to determine whether sepsis-induced immunosuppression persists. Herein, three-quarters of the patients have mHLA-DR below 15,000 AB/C at ICU discharge although they are usually free of organ failure at that moment. Furthermore, 12% of patients had mHLA-DR <5000 AB/C at ICU discharge, which represent a deep immune alteration. The immunological failure, not evaluated in routine, may represent a long-term persistent failure in the same way as renal failure in some patients. Among the three patients who died within 6 months post-ICU discharge, one died from cardiac arrest but two died from an infection and had abnormal mHLA-DR at ICU discharge. Interestingly, one of these infections was a CMV ileitis, which corresponds to a latent virus reactivation typically observed in context of deep immunosuppression.

With regard to adaptive immunity, CD4+ T lymphopenia occurring during sepsis is a major component of immunosuppression. It is also associated with higher risk of nosocomial infection and mortality [8, 26, 27]. Drewry et al. [8] reported that persistent lymphopenia on day 4 after the onset of sepsis predicted 28-mortality and 1-year survival. The medical literature on regulatory T cells is less abundant. Monneret et al. [12] reported in 2003 their relative increase in septic patients, which was later confirmed by others [28]. Their immunosuppressive properties affect both the antigen presenting cells of innate immunity and lymphocytes of adaptive immunity [29, 30]. Their presence correlates with a poor prognosis; in sepsis, their relative increase is observed early but persists only in patients dying later [12]. This negative association with survival was also reported in animal models [31]. We also recently reported that the increased proportion of Treg observed in sepsis was correlated with a decrease in lymphocyte proliferation [11]. Thus, Treg participates in the development of lymphocyte anergy described in sepsis which is significantly associated with increased mortality and the occurrence of secondary infections in septic patients [32]. In the present study, almost half of patients had CD4+ T lymphopenia on day 3–4 and this lymphopenia remained present for one-third of patients at ICU discharge although it was not correlated with the ICU length stay. The lack of correlation between immunological parameters and length of stay is probably caused by the small sample size resulting in a lack of power for correlation tests. Few patients (18%) had a percentage of Treg greater than 10%, but all of them had deep loss of mHLA-DR expression (i.e., mHLA-DR <5000 AB/C).

The study population was moderately old which may have affected immunological parameters. However, to our knowledge, modifications of mHLA-DR expression and CD4+ T cell count with age remain moderate [18, 33, 34]. Values found 6 months after ICU discharge were greater than the lower band of the normal range for all but one patient for mHLA-DR expression, and all but one for CD4+ T cell count. Thus, the immunosenescence does not seem to have an impact on the parameters measured.

With regard to immunological data at 6 months post-ICU discharge, the present results at the cellular level are in accordance with those from other investigators [35]. Arens et al. recently challenged the long-term persistence of sepsis-induced immunosuppression hypothesis in a retrospective cohort study composed of 8 sepsis survivors (without having studied immunological data during ICU stay, or at ICU discharge). They reported that immune alterations were minimal 26 months (range 9–52) after the septic event. There was no significant difference in lymphocyte profile (including Treg cell counts) and in expression of mHLA-DR between sepsis survivors and healthy volunteers. The expression of pattern recognition receptors (PRR) on leukocyte surface was not different between the two groups except for TLR-5, which was expressed at a lower level in the sepsis group. Finally, they only observed alterations regarding functional testing; after ex vivo stimulation of whole blood with potent activators, immune cells exhibited a decreased secretion of INF-γ, IL-10, IL-6, and TNF-α. Moreover, 62% of sepsis survivor patients had at least another episode of sepsis within the year prior to inclusion [35].

These findings raise the importance of functional tests, which must remain the reference to assess immune status as they directly measure the ability of cells to respond to pathogens. However, such tests are not always feasible in routine due to methodological difficulties such as a long incubation period, prolonged cellular purification procedures, and absence of standardization. Efforts are needed to facilitate practice of functional tests with the aim that their use becomes as common as that of static biomarkers.

The results presented herein complete two limitations of the study reported by Arens et al. [35]. Firstly, in the previous study, septic patients were not monitored during their ICU stay which means that sepsis-induced immunosuppression was not demonstrated in these patients. Secondly, the delay between septic event and mHLA-DR measurement was very long (median: 26 months), whereas herein it is demonstrated that mHLA-DR expression is already back to normal values as early as 6 months after ICU discharge. Additional studies with immunological monitoring within weeks following ICU discharge are needed to determine the timing of recovery of sepsis-induced immunosuppression.

Nevertheless, the present study has itself some limitations. For instance, only a small number of patients were studied at 6 months because a majority could not or refused to return to our hospital 6 months after the ICU discharge. Reasons for not coming back to the hospital were various. Most of these patients were elderly [median 71 (IQR 64–76) years (data not shown)], could not drive a car, could not take public transportation, and generally, they did not leave their home. This may have generated a selection bias in so far as participating patients were potentially in better clinical condition, which may have influenced results. We were not able to follow the immune status of patients with a bad outcome (those who died within 6 months or who suffered from new infections). This does not exclude the possibility that a sub-group of patients was not analyzed because of prolonged immune suppression. In other words, the analysis concerned only patients with a favorable outcome, whose immune functions returned to normal range. Data from the whole population are lacking, and these results need to be confirmed by a larger cohort. The small sample size also precludes a sub-analysis evaluating the immune status of patients suffering from complications in the long term in comparison with those who do not. However, these 15 patients were representative of the whole population, particularly for immunological parameters at ICU discharge. Thus, it should also be noted that 7.5% of patients alive at ICU discharge died within 6 months. Moreover, two other patients were out of contact and potentially also died. The total population had a favorable outcome because we included only patients who were alive at the time of ICU discharge, while mortality rate in septic shock is reported to reach up to 40% [1]. There is also a number of missing immunological data in particular at ICU discharge. This is due to the fact that measures could not be performed over the weekend or during the week outside the opening hours of the immunology laboratory. It would also have been interesting to have complete clinical data in particular the occurrence of infections since the ICU discharge for all patients who did not come back to the hospital 6 months after their ICU discharge. This could have allowed an exploration of a possible association with their immunological parameters at ICU discharge. Furthermore, we estimated only three immunological parameters, which also constitute a limit of the study.

If one hypothesizes that persisting long-term immune alterations may participate in increased risk for additional infections in septic patients, the present results are somehow unexpected. As opposed to mHLA-DR results, CD4+ T cells results and Treg results, the main result observed in the study reported by Arens et al. [35] is the ex vivo decreased whole blood cytokine secretion in long-term survivors. This is the confirmation that a single marker is likely to not be sufficient to fully monitor patient immune status. In addition, it supports the need to include functional testing in an immune monitoring panel which could detect some persistent immune alterations; additional research is needed to improve our understanding of long-term mortality in septic patients.

## Conclusions

This study did not find any persistent immune alterations in septic shock survivors with good clinical outcome 6 months after ICU discharge as evidenced by mHLA-DR expression, CD4+ T cells and Treg count. This does not exclude that immune alterations are present in a sub-group of patients with a bad clinical outcome. Nevertheless, CD4+ lymphopenia and low mHLA-DR expression were still present at ICU discharge for most patients.

## Abbreviations

mHLA-DR: expression of human leukocyte antigen DR on monocytes
Treg: regulatory T cells
ICU: intensive care unit
SOFA: Sepsis-related Organ Failure Assessment
IGS II: Simplified Index of Gravity II
AB/C: antibodies per cell
